# Retrieval Practices Enhance Computational and Scientific Thinking Skills

**DOI:** 10.3389/fpsyg.2022.892276

**Published:** 2022-06-29

**Authors:** Osman Yaşar, Jose Maliekal, Peter Veronesi, Leigh Little, Michael Meise, Ibrahim H. Yeter

**Affiliations:** ^1^The College at Brockport, State University of New York, Brockport, NY, United States; ^2^Rochester City School District, Joseph Wilson Magnet School, Rochester, NY, United States; ^3^National Institute of Education, Nanyang Technological University, Singapore, Singapore

**Keywords:** retrieval practices, computational thinking, scientific thinking, inductive reasoning, deductive reasoning

## Abstract

The notion of teaching experts’ habits of mind (e.g., computational thinking and scientific thinking) to novices seems to have inspired many educators and researchers worldwide. In particular, a great deal of efforts has been invested in computational thinking (CT) and its manifestations in different fields. However, there remain some troubling spots in CT education as far as how to teach it at different levels of education. The same argument applies to teaching scientific thinking (ST) skills. A remedy has been suggested to narrow CT and ST skillsets down to core cognitive competencies so they can be introduced in early and middle grades and continue to be nurtured during secondary and post-secondary years. Neuroscientists suggest that the *act of (computational) thinking* is strongly linked to the acts of information *storage/retrieval* by our brain. Plus, years of research have shown that retrieval practices promote not only knowledge retention but also inductive reasoning and deductive reasoning. Not surprisingly, these reasoning skills are core elements of both CT and ST skillsets. This article will mesh the findings of a teacher professional development with the existing literature to lay a claim that retrieval practices enhance CT and ST skills. The study offered training to secondary school teachers (*n* = 275) who conducted classroom action research to measure the impact of retrieval practices on teaching and learning of STEM and CT concepts. We used a quasi-experimental research design with purposeful sampling and a sequential mixed-methods approach focusing on the impact of professional development on teacher outcomes and, in turn, on student outcomes. A survey of teacher participants showed that the majority (96%) of survey respondents (*n* = 232) reported a good understanding of retrieval strategies, and how relevant ideas can be implemented and tested in the classroom. A large number of action research (target-control) studies by teachers (*n* = 122) showed that students who learned STEM and CS concepts through retrieval practices consistently scored 5–30% higher than those using the usual blocked practice. In most cases, the difference was statistically significant (*p* < 0.05). While the study contributes to retrieval practices literature, those looking for best practices to teach core CT and ST skills should benefit from it the most. The study concludes with some recommendations for future research based on the limitations of its current findings.

## Introduction

More than two decades ago, when computational science (an interdisciplinary practice incorporating modeling, simulation, visualization, and problem solving) emerged as a new workforce strategy for institutions of higher education (IHEs) and as an innovative teaching pedagogy for K-12, many had hoped that it would revolutionize the STEM education. As such, in 1998, SUNY College at Brockport launched the nation’s first undergraduate degree program in computational science (Yaşar et al., 2000; Yaşar and Landau, 2003; Turner et al., 2011). In 2006, Jeannette Wing, an influential computer scientist and an assistant director at the US National Science Foundation (NSF), mobilized significant NSF resources, rebranded computational science as computational thinking (CT), and claimed in her 2006 essay that CT should be taught as a fundamental skill in public schools just like reading and writing (Wing, 2006). The notion of teaching computational thinking (CT) as a fundamental competency seems to have inspired many educators and researchers worldwide. However, teaching experts’ habits of practice to novices is inherently problematic because of prerequisite content knowledge and practice skills needed to engage in the same thinking processes (Kirschner et al., 2006), not to mention the cost of providing them a similar environment to conduct inquiry and design. A remedy has been suggested to link experts’ habits of practice to fundamental cognitive processes so we can narrow their skillsets down to more basic competencies that can be taught to young students.

**Figure 1:**
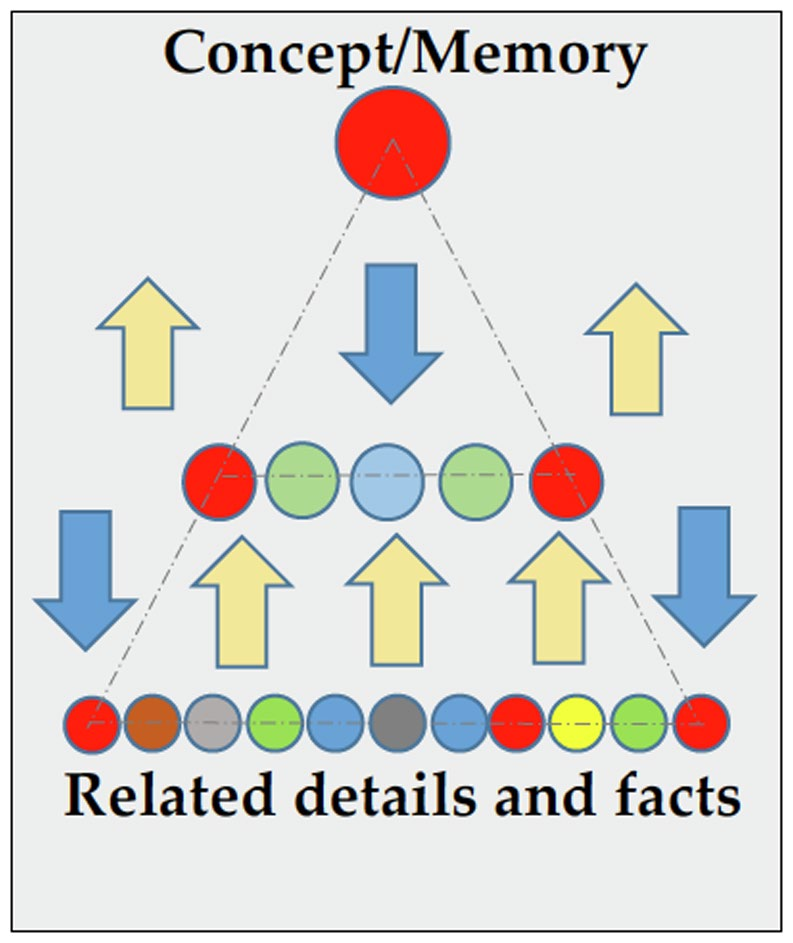
Distributive and associative ways of information storage and retrieval. Figure © 2017 IEEE. Reprinted, with permission, from (Yaşar, 2017).

Linking computation to cognition is not a new idea—in fact, it goes as far back as to the time of human computers during Babylonians (Denning and Tedre, 2019). Obviously, after the electronic computer age began in the 1940s, the term “computer” has often referred to electronic devices rather than human agents. What led to the design of electronic computing 80 years ago in the first place was that if thoughts (i.e., information) can be broken up into simple quantifiable constructs and algorithmic steps, then machines can add, subtract, or rearrange them as our brains do (Turing, 1936). The human brain employs a distributed network of neurons to rearrange information (Hebb, 1949). As such, information is stored into the memory *via* a specific pattern of neurons placed on a pathway and fired together. Arrival of new information lights up all related cues, neurons, and pathways in a distributive process that is similar to the top-down action in Figure 1, whereby a new concept is broken up into related pieces. The converse, retrieving information, involves reassembly of the original pattern of neurons and pathways in an associative process similar to the bottom-up action in Figure 1. Retrieval, in other words, is not an act of merely recalling facts and figures. It is a process of reassembly involving different pathways that are linked to one’s knowledge. What is retrieved is not a carbon copy of the original but a re-imagined copy of the original with some holes and/or extra bits. Neuroscientists see little or no distinction between the acts of information storage/retrieval and the act of (computational) thinking (Montague, 2006; Brown et al., 2014).

Our brain’s inclination to process information in an associative and distributive fashion, as well as to store and retrieve memories and concepts in a scatter and gather fashion by a distributed neural network, appears to be a manifestation of a basic duality engrained in the fabric of matter and information. Quantifiable things appear to behave in one of only two ways (as in Figure 1): they either unite associatively to form bigger constructs or break down distributively to smaller ones. Such a duality at the core of information processing by a computational mind carries itself up to higher-level cognitive processes, such as deductive reasoning in the form of distributive processing of information and inductive reasoning in the form of associative processing of information (Dunbar and Klahr, 2012; Yaşar, 2017, 2018).

We are all naturally inclined to employ inductive thinking and deductive thinking in everyday life. They are the two major cognitive competencies at the root of the CT skillset (Wing, 2006; Yaşar et al., 2016; Yaşar, 2018; Denning and Tedre, 2019; Mills et al., 2021), which are often cited as abstraction and decomposition skills. We all employ computational thinking by the virtue of having a computational mind. However, when used together in certain ways, the combination of inductive and deductive thinking becomes a much more powerful skill, as first described by Kant (1787) more than two centuries ago. For example, through *iterative* and *cyclical* use of inductive and deductive thinking, as depicted simplistically in Figure 1, does the conceptual change occur in our learning progression, all the way from childhood to the adulthood (Carey, 1985). Conceptual change is also at the heart of the scientific thinking (Vosniadou, 2013) both at the level of an individual scientist, or those who think like scientists, as well as that of the scientific progress by the scientific community (Kuhn, 1962; Thagard, 1999). Not surprisingly, imaging techniques have revealed that scientific thinking is not just thinking about the content (of sciences); it encompasses a set of cognitive processes, such as conceptual change, that transcend the field of science (Dunbar and Klahr, 2012). These processes include (a) problem solving, (b) design and modelling, (c) hypothesis testing, (d) concept formation, (e) conceptual change, and (f) reasoning (inductive, deductive, abductive, causal, and analogical thinking). According to Thagard (2012), these ST processes are no different from those employed in everyday living by non-scientists—the difference comes from how they are used. In a sense, what distinguishes ST from everyday thinking (i.e., computational thinking) is that while CT involves any use of inductive and deductive thinking, ST involves *iterative* and *cyclical* use of these two opposite reasoning skills to accomplish conceptual change and other ST skills listed above (Yaşar, 2021).

A great deal of efforts has gone into analyzing CT as a result of recent technological advancements which have affected our professional and personal lives. These efforts include definition of CT (Papert, 1980; Wing, 2006; Guzdial, 2008; Denning, 2009; Aho, 2012), its cognitive essence (Yaşar, 2017, 2018) and manifestations in different fields and ways to teach it at different levels of education (Denning, 2017a,b; Yadav et al., 2017; Denning and Tedre, 2019; Tedre and Denning, 2021). For a literature review, see Grover and Pea (2013); Angeli and Giannakos (2019); Denning and Tedre (2019); Kakavas and Ugolini (2019) and Saqr et al. (2021). In the 1990s, the focus was on literacy and fluency issues with a push to teach programming. The arrival of easy-to-use M&S tools, which hid the underlying mathematics and programming, allowed a new way of studying scientific phenomena and teaching CS principles in the 2000s. The present decade has seen even easier tools, such as mobile apps, to support children’s computational thinking and literacy skills (Papadakis, 2021).

Today, there are plenty of tools available for teaching various CT skills. However, the discourse on what it means to different stakeholders continues to this date. Some have suggested to categorize it as “CT for beginners” and “CT for professionals.” The same argument applies to teaching of ST skills. There is a need for innovative practices to provide continuity in CT and ST education all the way from elementary to post-secondary. We posit that an information processing approach to cognition, as briefly explained above, allows us to teach core CT/ST cognitive competencies with appropriate grade-level challenges and skills. If indeed the acts of information storage and retrieval strongly correlate to the act of computational and scientific thinking, then all we need to do is to strengthen those information processes. Whatever practices we come up with to strengthen them, one way to measuring their effectiveness could be through the act of information retrieval itself. We are lucky in that sense because long before such correlation was made, researchers in cognitive psychology had been studying the impact of memory retrieval practices on knowledge retention and other cognitive functions as explained in the next section. This article establishes ground that retrieval practices can be used as a way of strengthening CT and ST skills. We hope that the findings from our professional development program and related action research by participating teachers will shed a light on the discourse about CT and ST education. While the practitioners would benefit from reproducing similar results from a tested and scalable strategy, the researchers could expand their efficacy studies, *via* retrieval practices, to the teaching of more basic CT and scientific thinking (ST) concepts at a variety of grades. An approach such as the retrieval practice, which causes learning to stick and promotes core CT and ST skills, could have a broad impact in STEM education.

## Materials and Methods

### Teacher Professional Development

A grim situation occurring in most urban and rural school districts’ math and science achievement scores (National Center for Education Statistics, 1996; Qazi et al., 2020) has drawn concerns from local and state groups as well as the higher education institutions in our area. Educators point to poverty rates, lack of resources, and poor parental involvement as its root causes. Remedies suggested by State agencies include recruitment and training of effective leaders and teachers, ongoing professional development for teachers, maintaining standards, offering a rigorous curriculum for all, improving instruction *via* new technologies and pedagogies, and involving community; some of which have been adopted in our work, including the premise that, more than anything else, improving the teacher quality profiles would help improve STEM and CT education.

To support use of retrieval practices in secondary schools as an intervention, we offered and iteratively improved a 3-tier (beginner/intermediate/advanced) professional development (PD) to STEM teachers from partnering school districts in the area. The decision to offer a multi-tier program mainly came from: (a) our experience of previous PD effort (Yaşar et al., 2014) (b) recommendations by the ESC initiative at Los Angeles (Margolis et al., 2008; Goode and Margolis, 2011) (c) questions we got from districts to assure them continuing support and training, and (d) reports published by the Urban Institute (Beatriz, 2005) and others such as L-Horsley et al. (2010). The PD was based on the Iowa Chautauqua Learning Model (Blunck and Yager, 1996), with a summer institute and a series of academic-year training and debriefing workshops and mentoring activities. Attendance was voluntary, but project funds and school principals encouraged teachers to complete all 3 levels of training. Throughout the separate components of the PD, we used an expert-teacher-student cognition cycle and well-known principles of effective PD (Guskey, 2000; D-Hammond and Bransford, 2005; L-Horsley et al., 2010), including: (1) examining student work, (2) demonstration lessons, (3) lesson plans study, and (4) case discussions.

The beginner-level training trained participants on practicing tools (e.g., Google Forms and Microsoft Forms) and *spaced-out* retrieval strategies (see the next section for details of various retrieval practices mentioned here). Given the situation during the COVID-19 pandemic, we included online training as an option to increase the number of participants. The intermediate level training focused on (a) *distributed* retrieval practice, (b) use of the *rate of change* to model and simulate problems of interest (e.g., growth of disease and motion) with Excel, and (c) basics of conducting Action Research to improve classroom instruction (Ferrance, 2000). The advanced-level training included *generative* retrieval practices with simulations (SIMs) as well as basic programming skills (Scratch and Python). At the completion of the 3-year training, teacher participants were expected to learn and deploy various retrieval strategies, understand cognitive underpinnings of memory retrieval, CT, and ST, and conduct modeling and simulation of scientific phenomena using alternative tools (hands-on, Excel, web-based SIMs, Scratch, and Python). Summer teachers formed the backbone of the project, and their PD activity continued throughout the academic year in various forms to promote their skills to engage other teachers and help test and revise the resources for their colleagues and students. During the summer, returning teachers were asked to share evidence in the form of artifacts and presentations about a full year they left behind. Identifying parts of curriculum that are hard to teach where retrieval strategies might help was made a priority. We met the teachers where they were so they might build from their zone of proximal development (Vygotsky, 1978). Practice and peer sharing and critique throughout the school year were an integral part of this project. A sense of community and trust was nurtured *via* meetings, school visits, and online communications. We used a quasi-experimental research design with purposeful sampling and a sequential mixed-methods approach focusing on the impact of professional development on teacher outcomes and, in turn, on student outcomes. Details of the teacher PD program can be found in Yaşar et al. (2021).

### Retrieval Practices

Retrieval practices are grounded in research which suggests that new concepts are retained in memory far longer if the retrieval process is effortful, spaced-out, interleaved, and generative in nature (Brown et al., 2014). The empirical evidence further shows that (low-stakes) practice tests can serve as learning tools (Karpicke et al., 2014; Agarwal et al., 2020) to help students retrieve newly taught concepts in effortful ways that will, in essence, burn new knowledge into memory through connected understandings rather than rote memorization. A cognitive and constructive effort to recall recently learned concepts through connections to what had been previously stored in long-term memory has a much greater chance of being retained longer. If memory has lapsed, each time students engage in purposeful recall, it reassembles concepts through different pathways or links to one’s knowledge. One of the ways to accomplish these learning pathways is *spaced-out retrieval* (SR) practice through quizzes, self-testing, or flashcards. Spacing allows some forgetting that will trigger a cognitive effort for retrieval while repeated retrieval leads to more durable memories. Another one is *interleaving retrieval* (IR) practice that help link newly learned concepts to different contexts, changing conditions and parameters, and even multiple subjects. Often times, spaced-out and interleaving are used together and called *distributed retrieval* practice. A third one is *generative retrieval* (GR) practice; the act of trying to answer a question or attempting to solve a problem rather than being presented with an answer or the solution. The generative retrieval refers to both experiential and exploratory learning *via* trial-and-error. It is generally known to lead to complex mastery and greater knowledge of the interrelationships among parts of the problem and its solution. A learner may be able to arrive at the understanding of a phenomena on their own (Grabowski, 2004). One can practice generation by predicting an outcome or a concept before it happens while simply testing out their prediction and observing and noting the results—much like modeling and simulation (Yaşar and Maliekal, 2014).

These retrieval practices are all consistent with active learning and scaffolding strategies by which students are challenged in incremental steps as they build more skills (Mooney, 2013). The distributed retrieval (DR) practice has been tested in social sciences (Brown et al., 2014; Agarwal and Bain, 2019), math (Rohrer et al., 2014), natural sciences (Yaşar et al., 2019a,b, 2021; Samani and Pan, 2021), and computer sciences (Casanova et al., 2020) against the usual *blocked practice* whereby students learn to apply a certain method to solution of various questions of the same type on only one topic. An example of *blocked practice* would be to apply the Pythagorean Theorem to compute the hypotenuse of a right-angle triangle, *a*^2^ *+ b*^2^ *= c*^2^. Students need not learn to choose a solution method because problems within a blocked practice require the same strategy. In a *distributed* retrieval practice, two or more types of questions (and topics) are mixed as in Table 1, and students are faced with choosing a strategy to solve a problem. Despite the growing evidence about the impact of retrieval practices, they are yet to become prevalent in schools. The blocked practice is still the norm for many reasons, including a belief that repeated practice alone of the same drill builds up skills. The distributed practice does require a re-arrangement of topics within practices and lectures, but what is missing perhaps is a theory or framework to link retrieval practices to other educational reforms, such as CT, that are underway. This paper reports findings of a 3-tier teacher professional development, along with an extensive Action Research effort to examine the impact of distributed and generative retrieval practices on teaching and learning in secondary school STEM classrooms.

**Table 1:** An experimental set up to compare blocked vs. distributed practices.

Group	Assign #1	Assign #2	Time Delay 3 days	Unit Review & Test	Time Delay 15–30 days	Unannounced Test
Blocked 5 days	Topic X	Topic Y				
	Topic X	Topic Y				
Distributed 5 days	Topic Y	Topic X				
	Topic X	Topic Y				

Use of retrieval practices in the teaching of CT skills is very new. The only study that we have found in the literature is the one by Casanova et al. (2020) who examined if DR practices had durable effects on retention and learning of CT and programming concepts. A total of 10 elementary schools participated in a quasi-experimental study, consisting of 6 weekly sessions on CT concepts, including an introduction to Makey Makey (week 1), inequality symbols (week 2), identification and understanding of programming concepts such as conditions in week 3, loops in week 4, and inputs/outputs in week 5. A pre-questionnaire probed student familiarity and prior knowledge of Scratch, Makey Makey, and CT in general. Each session included hands-on practices with Scratch (Resnick et al., 2009; Funke et al., 2017) or Makey Makey (Silver et al., 2012), as well as DR practices (week 1 through 4) which involved quizzes made *via* Kahoot! with interleaved questions from current and preceding sessions. Students were given a review test at the end of the fifth session and an unannounced test a week later. The control condition and sample size were affected by COVID-19, and researchers used a *t-test* to compare scores of one group (*n* = 20) that consistently participated in all activities. While a week time may be short to test durability of concepts, the average score was higher for the unannounced test than the review test (72.6 vs. 67.9). The difference may not be all that significant (*p* = 0.19) but maintaining one’s score after 1 week is even a success. Test scores often go down over time due to loss of knowledge unless there is an intervention. There may have been other confounding factors such as students’ continuing exposure to topics in other units or similar courses.

## Results

### Teacher Professional Development

In a five-year timeframe (2016–2021), we trained 275 secondary school STEM teachers from 42 regional school districts (SDs), including 33% from urban, 30% from rural, and 36% from suburban SDs. 160 teachers returned for 2nd year advanced-level training, and of those, 40 teachers returned for 3rd year expert-level training. Yearlong support was offered to help deploy these strategies and conduct action research (Ferrance, 2000) in the classroom. Two independent evaluators were hired to design and conduct quantitative surveys and focus group interviews with teacher participants. One of these was Brockport Research Institute (BRI), which had previously conducted evaluations for over 60 National Science Foundation projects. BRI assigned two evaluation experts to design, collect, and analyze both teacher and student data. The other external evaluator was an education research faculty (Dr. John Tillotson) from a Teacher Education program at Syracuse University. While the BRI staff focused mainly on new participants who attended spaced-out and interleaved retrieval PD activities, Dr. Tillotson worked mainly with those returning to attend additional training on generative retrieval. Findings from both evaluation efforts took place simultaneously 3 years in a row and their overlap served as a triangulation of results and review of participant progress from distributed retrieval to generative retrieval. The teacher PD aspect of our program, along with its quantitative findings, has been described in an earlier publication (Yaşar et al., 2021). Below, we will only give a brief account.

In quantitative teacher surveys (see Table 2) conducted annually between 2017 and 2018, scores above 4.0 on a 5-point scale for nearly every survey item suggested that teachers found the program to be both engaging and effective in raising their awareness of retrieval strategies and of the research base supporting their efficacies within STEM classrooms as indicated by mean. The respondents highly valued the opportunities to collaborate with other teachers in designing online retrieval practices and considering possible research designs for studies they planned to conduct during the upcoming school year. Ninety-six percent (96%) of the teacher respondents highly valued the opportunity to learn about distributed retrieval strategies and the research base supporting the effectiveness of this pedagogical approach in K-12 classrooms. A majority of the teacher participants (96%) also indicated that the time devoted to accomplishing each of the primary workshop objectives was appropriate and that the workshops were effective in helping them develop a clear understanding of their roles and responsibilities pertaining to the classroom-based research component of the initiative (93%).

**Table 2:** Likert-scaled questions and answers from summer workshops.

The following questions apply to spaced-out (SR), interleaved (IR), and generative (GR) retrieval strategies/practices as indicated by enclosed within brackets.	Score (out of 5)		
	2017 SR *n* = 10	2018 IR *n* = 26	2019 GR *n* = 18
1. The summer workshop dates and times fit well with my schedule and commitments.	4.8	4.72	4.94
2. The goals and expectations were clearly articulated by the project leadership team.	4.9	4.16	4.94
3. The time devoted to accomplishing each of the primary objectives was appropriate.	4.9	4.36	4.88
4. After the workshop I now have a deeper conceptual understanding of the research and literature supporting the use of [SR/IR/GR] retrieval practices in the classroom.	4.9	4.56	4.82
5. The workshop enhanced my skills in using digital devices, relevant tools [SIMs], and mobile Apps to assess students’ understanding of important concepts.	3.7	3.96	4.82
6. The workshop increased my confidence and ability to design classroom-based research to investigate the effectiveness of [SR/IR/GR] retrieval practices	4.5	4.24	4.60
7. The workshop provided me a chance to interact with colleagues to discuss the use of SIMs and digital mobile App development ideas and potential research projects	4.6	4.08	4.92
8. The project overview, leadership, and framework discussion for the research needs was effective in helping me understand my role and responsibilities as a participant	4.4	4.24	4.60
9. The workshop was effective in helping me design Action Research cycles to test the impact of SIM-based generative retrieval on my students’ learning outcomes.	NA	NA	4.88
10. Overall, the workshop was effective in preparing me with the knowledge and skills necessary to successfully participate in the project during the upcoming school year.	4.7	4.32	4.88

In another subsequent quantitative teacher survey conducted after the 2019 summer workshop on the generative retrieval strategy and related deployment, the data shown in Table 2 suggest that the workshop gave them a good understanding of the generative retrieval practice, enhanced their skills to use SIMs and mobile apps, increased their confidence and ability to design action research, provided a chance to interact with colleagues, and helped them design an experiment to measure the impact of SIM-based generative retrieval. All of participating teacher respondents (100%) found the workshops to be valuable overall (strongly agreed or agreed). More specifically, they all indicated that the workshops were effective in providing them with the conceptual knowledge and practical skills necessary to effectively engage students in SIM-based generative retrieval during the 2019–2020 school year. Similarly, 92% of the STEM teacher respondents highly valued the opportunity to learn about generative processes and the research base supporting the effectiveness of these pedagogical approaches in K-12 classrooms. All teacher participants indicated that the workshop prepared them with knowledge and skills to successfully design, implement, and even conduct classroom-based action research project to assess the impact of intervention on students’ learning outcomes. The surveys’ construct and face validities had been confirmed by their designers and the experts they hired.

The surveys were followed up by an enriched qualitative case study (focus group interviews) to explore the meaning of trends/findings in the quantitative part of the study. Two independent experts coded the responses and used an inductive process (Creswell, 2012) to form broader themes. For example, one of the broad themes was that the workshops afforded teachers deeper insight into the practical aspects of the classroom implementation of retrieval strategies and the positive effects research has shown these techniques to have on student learning outcomes. Another theme was that workshops provided ample time for the participants to delve deeply into their work in creating the required SIM-based learning exercises that would be implemented in the following school year. Evaluators used several forms of validation, including triangulation *via* data from multiple sources and member checking by asking project teachers and faculty to review the findings (Fincher and Petre, 2005). Further details of qualitative findings are being published separately due to their nature and volume. The main focus of our article here is on classroom action research by teachers, which will be given in the following two subsections.

### Distributed Retrieval Practice

A large cadre (*n* = 82) of trained teachers participated in the Action Research to explore the impact of distributed retrieval (DR) practices (the combination of spaced-out and interleaving) in teaching and learning of various topics in geometry (e.g., quadrilaterals and altitudes in right-angled triangles), biology (photosynthesis, respiration, and Punnett squares), chemistry (Le Chatelier’s Principle, Potential Energy Diagrams, heat, and half-life decay), and earth sciences (erosion and planetary motion). Of these, 68 teachers conducted research 3 years in a row using different classes and student populations while improving their methods of selecting topics and students more randomly and increasing the delay time between pre- and post-assessments. Google (and Microsoft) Forms provided a framework by which students could use a mobile program to record their thinking while the assessment data could be collected by the teacher and ultimately shared with students for immediate feedback. The science teachers used a question bank (e.g., CastleLearning™) to draw questions from. Students were placed randomly into control and target groups of equal sizes ranging from 12 to 32 depending on each study. About half the cases followed a research design whereby one group (A) followed the blocked (BL) strategy while the other group (B) used a DR strategy for practices and assignments. Other cases followed a design whereby each group (A and B) was taught using both strategies (blocked and distributed) alternately, though care was taken to make sure that group placement was not visible to students—that is, all students participated in simultaneous classroom instruction. Instruction for each topic lasted for the same number of school days with both strategies. An in-class review of both topics was conducted a short while after completion of instruction. The review was concluded with a test which in some cases served as a baseline. Finally, an unannounced test was conducted later to measure student retention of the content knowledge 15–30 days after the review. In most cases, teachers conducted pre- and post-activity assessments with multiple-choice questions on all control and target groups to identify and reduce the number of confounding variables and triangulate the results as much as possible.

In 82 independently run control-target experiments, like the one in Table 1, students who learned STEM and CS concepts through DR practices scored better than those using blocked (BL) practice in 80 cases (95% of the time). The average of pre- and post-scores for DR groups was 70.7 (pre) and 71.1 (post), meaning that knowledge was retained, whereas the average scores were 70 (pre) and 65 (post) for BL groups, meaning information was lost (*p* < 0.007), during the blocked practice. The difference between DR & BL post-assessment averages (71.1 vs. 65) was also significant (*p* < 0.015). Standard deviation in group average scores was 15 (pre) and 16 (post) for BL and 15 (pre) and 12 (post) for the DR groups, implying more consistency in post-assessment scores by the DR groups. A few representative studies are shown below to illustrate more details. *T*-test statistics was used to examine the significance of differences both between and within groups.

- 10th Grade Chemistry (Topics: Half-life, Heat formula): While Group A (*n* = 20, blocked) and Group B (*n* = 18, distributed retrieval) scored about the same (Group A at 63.5 vs. Group B at 61.4) at the review (pre) test for half-life, their performance on an unannounced test given 30 days later differed significantly (*p* = 0.014) with Group A’s average being 40 vs. Group B’s 63.3. On the topic of heat, while Group A scored significantly (*p* = 0.027) higher than Group B (81.1 vs. 68.8) at the review test, its performance (32.6) on an unannounced test given 30 days later fell substantially below Group B’s (42.4). The drops in performance by both groups were statistically significant (*p* < 0.0006), yet the drop by the group with the blocked practice was twice as high as the drop by the group with the distributed retrieval practice.
- 9th Grade Chemistry (Topics: Photosynthesis, Respiration): Two groups (*n* = 22 each) experimented with alternating practices on different topics. While Group A used the blocked practice to learn about photosynthesis, Group B used the distributed retrieval practice. Similarly, while Group B used the blocked practice to learn about respiration, Group A used the distributed retrieval practice. Groups were compared to themselves (pre-test vs. post-test) to see their self-improvement and to their counterparts (post-tests) which learned the same topic *via* different practices. As shown in Table 3, while groups that used the blocked practice scored higher on pre-tests, they scored lower than the groups with the distributed practice. While pre- and post-test differences between groups are not statistically significant, the improvements by the distributed groups from pre-test to post-test are significant and large enough to exceed their counterpart.
- 9th Grade Technology Education (Topics: Design, Drawing, and Production). Group A (*n* = 17, blocked practice) and Group B (*n* = 17, distributed retrieval practice) scored 65.6 and 72.5 on the pre-delay test. 30 days later, the score for the group with distributed retrieval remained about the same (76.25) while the score for the group with blocked practice went down significantly (*p* = 0.04) to 56.87.
- 7th Grade Science (Unit: Motion, Topic: Acceleration). Group A (*n* = 21) and Group B (*n* = 20) were taught acceleration by the same teacher. 15 days later, both groups received a unit review, followed by a test to set a baseline for the recall later. As shown in Table 4, both groups scored the same right after the unit review. In an unannounced test 15 days later, however, the average score for the group (A) with blocked practice decreased significantly (*p* < 0.01) by 22.8%, whereas the average score for the group with distributed retrieval (B) decreased by only 5.5%, indicating that retrieval practices helped students retain knowledge better than the blocked practice.
- 7th Grade Biology (Topic: Punnett squares): The average score of 4 daily assignments conducted in the same week by Group A (blocked, *n* = 27) and Group B (distributed retrieval, *n* = 29) were about the same (46.79 vs. 47.34 out of 100). As shown in Table 5, the average scores on the review test were 55.88 (Group A) and 52.45 (Group B). When an unannounced quiz was given to both groups 15 days after the review test, Group B not only retained its knowledge of the topic but outscored Group A while improving its average significantly by 8 points (*p* < 0.01) to 60.21. Group A scored slightly better with 2-points compared to its review test. A similar trend was seen in the analysis of each student’s progress for both groups: 19 students in the distributed retrieval group increased their score while 8 decreased versus 14 increasing and 10 decreasing in the blocked group.
- 7th Grade (Topics: Erosion, Planetary Motion): As shown in the Table 6, Group A (distributed, *n* = 30) outperformed Group B (blocked, *n* = 31) by 9% on a post-test on weathering and erosion; a difference that is statistically significant. In another experiment on planetary motion and the effect of mass on the gravity of an object, Group B (distributed, *n* = 21) outperformed Group A (blocked, *n* = 29) by 38%; a difference that is also statistically significant. In the same experiment, the distributed group outperformed the blocked group by 30% on levels of organization (progression of levels by cell(s) to reach an organism).

**Table 3:** Comparing 9th grade chemistry classes using blocked practice vs distributed retrieval practice.

	Group A *n* = 22	Group B *n* = 22	*Is the difference statistically significant?*
Topic: Photosynthesis	BLOCKED	DISTRIBUTED	
Pre-test	53.66	45.86	No (*p* = 0.19)
Post-test	57.47	62.04	No (*p* = 0.55)
*Is the difference significant?*	No (*p* = 0.59)	Yes (*p* = 0.001)	
**Topic: Respiration**	**DISTRIBUTED**	**BLOCKED**	
Review test	15.59	20.27	No (*p* = 0.14)
Post-review test	57.72	50.90	No (*p* = 0.33)
*Is the difference significant?*	Yes (*p* < 0.01)	Yes (*p* < 0.01)	

**Table 4:** Comparing 7th grade science classes using blocked practice vs distributed retrieval practice.

Topic: Motion	Group A, *n* = 21 BLOCKED	Group B, *n* = 20 DISTRIBUTED	*Is the difference statistically significant?*
Pre-test	70	72	No (*p* > 0.05)
Post-test	54	68	Yes (*p* < 0.01)
*Is the difference significant?*	Yes, *p* < 0.01	No, *p* > 0.05	

**Table 5:** Comparing 7th grade biology classes using blocked practice vs distributed retrieval practice.

Topic: Punnett Squares	Group A (*n* = 27) BLOCKED	Group B (*n* = 29) DISTRIBUTED	*Is the difference statistically significant?*
Review test	55.88	52.45	No (*p* = 0.52)
Post-test	57.67	60.21	No (*p* = 0.71)
*Is the difference significant?*	No, *p* = 0.50	Yes, *p* = 0.01	

**Table 6:** Comparing 7th grade earth sciences classes using blocked practice vs distributed retrieval practice.

Topic ↓	Post-test (Group A)	Post-test (Group B)	*Is the difference significant?*
Erosion	**DISTRIBUTED (***n* **= 30)** 81.66	**BLOCKED (***n* **= 31)** 75.16	yes; *p* = 0.013
Planetary motion	**BLOCKED (***n* **= 29)** 51.13	**DISTRIBUTED (***n* **= 21)** 70.5	yes; *p* = 0.008

### Generative Retrieval Practice

With a vast array of simulations (SIMs) available (in-house and on the internet) as surrogates for real phenomena, student comprehension of a STEM topic or phenomena was compared using generative retrieval practices with SIMs against regular practice with text and illustrations. Neither group had been previously introduced to topic-related concepts whose understanding was the purpose of this phenomena-first experiment. Secondary school teachers (*n* = 40) in this study selected up to 6 topics to compare learning in control-target groups using 3-level Socratic level questions (based on Bloom’s Taxonomy), testing for growing complexity, interrelationships, and greater content knowledge (see Table 7). Level 4 and higher were optional. While students were not randomly selected (they came from each teachers’ classes), the groups were reversed for half of the topics, so each group had access to equal number simulations and illustrative texts. This eliminated the concern that one group was simply academically superior to the other group. T-test statistics was used to compare group scores.

**Table 7:** A template for socratic questions in generative retrieval practices.

Overall Goal	Construct a minimum of 12 questions per simulation (SIM), including illustrated text. NOTE: Levels 4 and up are optional.
Level 1	Explore students’ first thoughts and observations; clarify student’s thinking
Level 2	Challenge student thinking; have students manipulate the SIM to challenge such thinking
Level 3	Point out the evidence; ask for evidence that backs up student claims
Level 4	Point out counter thinking; ask students for conflicting issues, if any
Level 5	Explore student expertise of the concept/phenomena; ask “if/then what happens” questions
Level 6	Question the intent of questions asked; Explore the main idea of the simulation

In order to generalize the findings, we combined data across multiple subjects, specific topics, teachers, school districts, etc. to remove confounding variables. Of the ~14,000 questions answered by control and target group students, students in the generative SIMs practice consistently answered more questions correctly. The comparison test was run at all question levels at a significance level of 0.05. As expected, performance decreased in both groups as the level of the question increased, but the drop-off was more pronounced in the illustrative text group (18% worse on level 3 vs. level 1) than in the generative retrieval group with SIMs (7% worse on level 3 vs. level 1). Target group students answered level-1 questions 2% more correctly and the difference rose to 5% more correctly for level-2 and 8% more correctly for level-3 questions. In all levels, the *value of p* was less than 4×10^−4^. The standard deviation was also smaller for the generative group, implying more consistency in its results. Students in the generative retrieval group not only performed better but also SIMs-based generative retrieval was superior as a delivery method to increase comprehension of STEM and CT concepts as well as critical thinking. A few representative studies are shown below to illustrate more details.

- 10th Grade Special Education/Algebra: (Topics: Graphing Quadratics, Vertex Form of Quadratic Equations, Point Slope, Slope Intercept, Solving Linear Equations with One Variable, Quadratic Solutions, *n* = 81). Research Design: Random division of the class populations from two Intro to Algebra courses and three Algebra/Common Core courses with both General Education and Special Education students. In both control and target groups, there were 81 students; of which 43 students were special education students. As shown in Table 8, the groups with SIM generative retrieval practice overwhelmingly outperformed the groups with text illustrations. According to the teacher report, the SIMs allowed students to use visual examples and check scenarios to come up with correct answers more quickly than other students who used text and illustrations. In particular, this kind of visual and interactive aspects of practice helped special education students more significantly because they had reading levels below their grade level.
- 9th Grade Biology (Topics: Circulation, Homeostasis, Enzymes, Photosynthesis, Diffusion, Natural Selection, and Gene Expression, *n* = 15). Since students were not allowed to use the internet without supervision and since they have limited access to computers, the teacher decided to display the simulations on the Smart Board and allowed students to do the practice as a group exercise. According to the teacher report, students showed a fear of failure with the first couple of SIMs even when they were told that this would not affect their grade. Compared to the text-based exercise, they answered more questions correctly. While this was not readily apparent for level 1 questions, it became more noticeable for the level 2 and 3 questions. The group aggregate over the use of multiple SIMs is also given in Table 9.
- 8th Grade Biology (Topics: Circulation, Homeostasis, Enzymes, Photosynthesis, and Diffusion, *n* = 30). Research Design: Group A had SIMs for topics 1–3 and Text with Illustrations for topics 4–5 while Group B had Text with Illustrations for topics 1–3 and SIMs for topics 4–5. Each group had topic-related level-based questions, which generally included 6 to 8 level-1 questions, 2 to 3 level-2 questions, and 1 to 3 level-3 questions. As shown in Table 10, the groups with SIMs scored higher at all levels except for topic 2 (homeostasis). However, as questions got more difficult and complicated, even for homeostasis (#2), the group with SIMs scored higher.
- 8–11th Grades General Science and Physics: (Topics: Forces on a Ramp, States of Matter, Ideal Gas Law, Pendulums, Projectile Motion, and Hooke’s Law, *n* = 68). Research Design: A heterogeneous population of 68 students, ranging in age from 12 to 18 in different classes (8th grade General Science and 11th grade Regent Physics). Students were randomly divided in half for each of the 6 modules. While one group practiced *via* phenomena first (SIMs), the other practiced *via* traditional (Text) instruction using text and illustrations. As shown in Table 11, groups with SIMs generally outperformed the other groups with text and illustrations. However, the difference is significant for only half of the topics.
- 6th Grade Introductory Computer Programming (Topics: Flashing Heart, Name Tag, Coin Flip, Smiley Face, Random Dice, Rock Paper Scissor, *n* = 30). Research design included having two different classes each doing 3 SIMs and 3 Text with Illustrations. As shown in Table 12, groups with SIMs consistently and significantly outscored others. The teacher used Microsoft MakeCode (a free open-source platform) for creating engaging CS learning experiences that support a progression path into real-world programming. He followed Project Lead the Way’s Computer Science curriculum and repeated the experiment twice (Fall and Spring) with two different samples. He noted that students appeared to have put more effort into completing the SIMs during Spring 2020 COVID lockdown and focused more on the questions rather than relying on help from the teacher.

**Table 8:** Comparing 10th grade math classes using SIM-based generative practices vs text-based illustration practices.

**(a)**													
Topics ➔	Point slope						Slope intercept						
Students ➔	All students		General edu.		Special edu.		All students		General edu.		Special edu.		
Practice ➔	SIM	Text	SIM	Text	SIM	Text	SIM	Text	SIM	Text	SIM	Text	
Level1	56	25	80	41	32	10	72	55	83	80	61	31	
Level2	30	17	41	27	19	8	34	21	60	31	8	11	
Level3	38	8	39	11	38	5	33	20	38	29	28	11	
Total	46	20	62	32	30	8	53	37	66	54	40	21	
**(b)**	**Graphing quadratics**						**Vertex form of quadratic functions**						
Level1	64	32	70	48	58	16	58	35	74	54	42	17	
Level2	27	22	40	35	15	10	51	32	67	50	36	16	
Level3	28	10	41	17	15	3	32	18	49	27	15	10	
Total	51	28	60	42	42	14	54	33	70	50	38	16	
**(c)**	**Solving linear equations**						**Quadratic solutions**						
Level1	76	62	88	74	65	49	42	25	47	31	37	18	
Level2	31	22	41	30	21	14	28	16	38	21	17	11	
Level3	21	10	26	18	16	2	19	5	29	6	9	4	
Total	36	25	44	34	28	16	32	17	40	22	25	13	

**Table 9:** Comparing 9th grade biology classes using SIM-based generative practices vs text-based illustration practices.

Topics ➔	Circulation		Homeostasis		Enzymes		Photosynthesis		Diffusion		Total	
Method ➔	SIM	Text	SIM	Text	SIM	Text	SIM	Text	SIM	Text	SIM	Text
Level1	100	66	100	83	100	0.0	100	100	80	80	96	64
Level2	80	50	50	50	100	0	75	100	66	0	76	58
Level3	100	50	50	0.0	66	100	100	66	75	75	80	60
Total	93	60	80	60	90	20	91	90	73	70	85	61

**Table 10:** Comparing 8th grade biology classes using SIM-based generative practices vs text-based illustration practices.

Topics ➔	Circulation		Homeostasis		Enzymes		Photosynthesis		Diffusion		Total	
	SIM	Text	SIM	Text	SIM	Text	SIM	Text	SIM	Text	SIM	Text
Level1	86	75	72	89	89	89	78	64	87	68	85	79
Level2	45	37	94	95	79	80	83	70	91	71	75	70
Level3	21	11	59	56	51	26	82	61	63	14	54	42
Total	72	62	76	85	84	82	80	65	85	51	78	72

**Table 11:** Comparing grades 8–11 general science and physics classes using SIM-based generative practices vs text-based illustration practices.

Topics ➔	Forces on a ramp		States of matter		Ideal gas law		Pendulums		Projectile motion		Hooke’s law		Weighted average	
Method	SIM	Text	SIM	Text	SIM	Text	SIM	Text	SIM	Text	SIM	Text	SIM	Text
Size	7	9	16	15	16	18	15	15	18	16	15	17		
Level1	96	83	74	51	72	73	67	67	94	86	75	61	78.50	69.37
Level2	86	67	62	53	81	71	36	55	64	61	62	51	63.35	59.37
Level3	79	44	75	60	80	79	47	32	56	61	56	24	64.20	50.91
Total	89	69	71	53	77	74	53	56	76	72	65	43	70.44	60.78

**Table 12:** Comparing 6th grade introductory computer programming classes using SIM-based generative practices vs text-based illustration practices.

Topics ➔	Flashing heart		Name tag		Coin flop		Smiley face		Random dice		Rock paper scissor		Weighted average	
**Fall**	SIM	Text	SIM	Text	SIM	Text	SIM	Text	SIM	Text	SIM	Text	SIM	Text
Level1	100	75	100	100	100	100	100	66	100	100	100	66	100	82.3
Level2	66	33	100	75	50	25	66	33	75	25	60	20	69.5	34.7
Level3	66	66	100	66	75	50	75	25	75	50	50	50	75	50
Total	80	60	100	80	70	50	80	40	80	50	70	40	80	53.3
**Spring**														
Level 1	100	50	100	66	100	50	100	66	100	50	100	66	100	58
Level 2	66	33	75	25	75	25	100	66	75	50	80	40	78	39
Level 3	100	33	100	0	50	50	75	25	75	75	100	50	80	40
Total	90	40	90	30	70	40	90	50	80	60	90	50	85	45

## Discussion

To support new pedagogical experiences, we offered professional development opportunities on memory retrieval strategies to secondary school teachers from an urban city surrounded by many suburban and rural school districts. Both quantitative and qualitative data from participating teachers pointed to the effectiveness of the spaced-out, interleaved, and generative retrieval strategies in the classroom. The multi-year quantitative and qualitative data consistently suggested that all of the participating teachers found the workshops to be valuable overall, specifically in providing them with the conceptual knowledge and practical skills necessary to effectively engage in implementation efforts during the school year. Similarly, they highly valued the opportunity to learn about generative processes and the research base supporting the effectiveness of these pedagogical approaches in K-12 classrooms.

An overall analysis of student data from classroom action research studies shows that those who learned a topic *via* the distributed retrieval strategy scored considerably better than those who learned in the traditional (blocked) way. In many cases reported, the differences were statistically significant in favor of the distributed retrieval practice. While students in the DR practice group retained knowledge of the topics, students in the BL practice group lost on the average 8% of it after 2 weeks. For experiments with longer time delays (30 days), the knowledge loss reached up to 60% (for example, see the first bullet listed in the Distributed Retrieval Practice section above). A drop in performance after 30 days is normal for both groups because of natural information loss due to time, but in some cases the drop for the blocked practice group was twice as high as the drop by the treatment group.

A similar trend was seen with students who learned a concept *via* a phenomena-first approach through interactive and generative retrieval using modeling and simulations (M&S) versus others who were first lectured about the topic through reading text and illustrations. This is consistent with the literature on the use of modeling and simulation in science education (Stinken-Rösner, 2020). Researchers have reported that M&S supports both inductive and deductive approaches to learning (Rutten et al., 2012; Smetana and Bell, 2012; Yaşar and Maliekal, 2014). Teachers have historically used deductive pedagogies to instruct students (Bransford et al., 2000). In this approach, information flows from general to details (top-down), or from simple to more complex, as seen in Figure 1. The teacher introduces a concept and then shows supporting facts, applications, and details to afford students an opportunity to apply it themselves. In an inductive pedagogical approach, the flow of information is from details to more general (bottom-up) and it is the student, not teacher, at the center of action. Through experimentation and problem solving, students are led to discover their own conclusions by sorting out details and connecting the dots to arrive at more general patterns and principles. Inquiry-based teaching is one such form. Since learning invariably involves movement in both directions (Haight et al., 2007), a teaching that matches bi-directional learning could maximize the benefits (Prince and Felder, 2007).

Limitations of our study include some confounding variables such as suitability of content for distributed and generative practice, and each group’s prior experience and background were possibly in play in our research. We suspect that the level of improvement in some cases depended on the nature of topics, grade level, as well as teacher experience and school environment. For example, mathematics and geometry seem to be well suited for retrieval practices. The benefits to students with special needs (as seen in Table 8) seemed greater in all content areas tested by one of the best math teachers in the program. However, given the large number of cases, both the distributed and generative retrieval practice outshined the traditional blocked practice.

Our findings complement those in the literature about the impact of retrieval practices on retention (Brown et al., 2014; Agarwal and Bain, 2019) as well as on comprehension of STEM (Rohrer et al., 2014) and CT concepts (Casanova et al., 2020). In almost all cases, students in the treatment groups not only seemed to retain their knowledge of the topics they were tested on, but they also improved their scores, an indication that retrieval helped them comprehend the topics better and make inductive and deductive associations with other topics taught within the same course. This is especially true for the groups with generative retrieval practices because students’ ability to comprehend a topic was put to test without their exposure to the material about such topic ahead of time. The effectiveness of SIM-based generative retrieval may be partly driven by inductive and deductive cognitive processes of modeling and simulation, but other researchers have also reported that retrieval practices support inductive and deductive learning even without the use of modeling and simulation tools. While we did not directly measure the impact of SIM-based generative retrieval on basic reasoning skills (i.e., inductive and deductive thinking), recent reviews by Birnbaum et al. (2013); Brunmair and Richter (2019), and Firth et al. (2021) cite that interleaving retrieval practices improve inductive learning—a mental process of acquiring conceptual knowledge from the synthesis of exemplars (Prince and Felder, 2007) that is often known as abstraction in both CT and ST literatures (Wing, 2006; Dunbar and Klahr, 2012; Thagard, 2012; Yaşar et al., 2017; Denning and Tedre, 2019). Wissman et al. (2018) found spaced-out retrieval to improve deductive learning—a mental process of testing factual knowledge, a formula, concept, or theory to various scenarios (Prince and Felder, 2007) as described earlier in terms of distributive processing of information. Eglington and Kang (2018) also reported that retrieval practice improves deductive inference (*p* = 0.013, d = 0.41). Deductive thinking skills are often categorized as decomposition skills in the CT literature (Wing, 2006; Barr and Stephenson, 2011; Denning and Tedre, 2019) and as analysis skills (the opposite of synthesis) in the ST literature (Dunbar and Klahr, 2012; Thagard, 2012). According to Eglington and Kang (2018)—Kang was a collaborator of the PI on and NSF project—for the spaced-out retrieval practice to benefit students’ deductive thinking, the material to draw appropriate inferences from may need to be presented together.

There is strong evidence that the combination of spaced-out and interleaving retrieval (called distributed retrieval) practices promotes both inductive and deductive thinking skills—which, when used in an iterative and cyclical fashion, constitute a major thrust of ST (Dunbar and Klahr, 2012). Additionally, Samani and Pan (2021) examined their effects on factual knowledge and problem-solving skills. Over 8 weeks, students (*n* = 350) in two lecture sections of an introductory physics course practiced interleaving in thrice-weekly homework assignments. The control group practiced one topic at a time whereas the target group practiced alternating topics. The study consisted of two stages, similar to those we reported, where in stage 1 class A used blocked practice and class B used interleaved practice, and in stage 2 class A used interleaved practice and class B used blocked practice. In each of the two stages, students completed 84 practice problems across 10 homework assignments. On two unannounced tests (one at the end of each stage) containing novel and more challenging problems than those in the homework assignments, the target group recalled more relevant information and more frequently produced correct solutions (with observed median improvements of 50% on test 1 and 125% on test 2). Effect sizes were reported in terms of Cohen’s d. Interleaving retrieval yielded higher test scores than blocked practice in Stage 1 (*d* = 0.40, *p* = 0.0008) and Stage 2 (*d* = 0.91, *p* < 0.0001). When spaced-out, even restudy or cramming (blocked practice) has its benefits in terms of helping students to apply (or transfer) the same formula or facts to the solutions of various problems or situations. Karpicke and Blunt (2011) and others (Butler, 2010; Rohrer et al., 2010) showed that repeated retrieval practice of scientific concepts could promote transfer to test questions which are related to, but different from, originally practiced materials.

## Implications, Conclusion, and Recommendations

The findings of classroom action research by a large number of teachers in our study support the use of retrieval practices for retention and comprehension of secondary school STEM and CT concepts. This is consistent with the literature on retrieval practices (Agarwal et al., 2020). More importantly, our study complements findings of other recent studies such as Prince and Felder (2007), Birnbaum et al. (2013), Eglington and Kang (2018); Brunmair and Richter (2019); Firth et al. (2021), and Samani and Pan (2021) to point out that retrieval practices enhance core elements of computational and scientific thinking. While other researchers had reported a favorable impact of retrieval practices on inductive and deductive thinking, a more general realization that—taken together with our findings—retrieval practices enhance core CT and ST skills is the most important conclusion of our study. This is a timely discovery because of the ongoing search in the STEM community for innovative and fundamental practices and tools to support CT and ST education across different levels of education (Denning and Tedre, 2019; Papadakis, 2021). The authors posit that use of retrieval practices is perhaps one of the most direct ways of improving core CT skills because memory retrieval is nothing but the thinking itself by a computational brain. For those who are looking for best practices to improve CT and ST skills of young children, memory retrieval practices set a natural example.

There were several limitations of our study, which can be improved in future studies. Even though a large number of action research studies (*n* = 122) by teachers consistently point to the benefit of retrieval practices, some confounding variables such as suitability of content for retrieval practices as well as prior experience and background by control and target groups were possibly in play in our research. Circumstances surrounding each experiment were obviously different because they were each run by different researchers. This may have also been good to eliminate bias. At the same time, we suspect that the level of improvement in some cases depended on the nature of topics, grade level, as well as teacher experience and school environment. The control variables can be isolated better in future studies. Also, the time delay between the unannounced test and the review test (the last time students are exposed to the topic) should be long enough (at least 30 days) to allow more contrast, if any, to surface out between control and target groups. In some cases, it appears that performance of both groups was higher at the final test than the review test, which means the examined topics were continued to be discussed either in similar classes that students attended or in the same class within the context of other topics. In studies where group exposure to the examined topic were better isolated, student retention appears to go down for both groups but more so for the group with blocked practice than the group with retrieval practices. Finally, since the current study was limited to secondary schools, the authors recommend expanding the study to lower grades. Future research should include use of retrieval practices in a wider set of CT and ST concepts. A recommendation for the computer science education community is that because of the importance of programming in CT education, there is a need for more studies such as Casanova et al. (2020) to examine effect of retrieval practices on retention and learning of programming concepts.

## Data Availability Statement

The raw data supporting the conclusions of this article will be made available by the authors, without undue reservation.

## Author Contributions

OY wrote the manuscript and led the research. All authors helped to conduct the research and implement that program. All authors contributed to the article and approved the submitted version.

## Conflict of Interest

The authors declare that the research was conducted in the absence of any commercial or financial relationships that could be construed as a potential conflict of interest.

## Publisher’s Note

All claims expressed in this article are solely those of the authors and do not necessarily represent those of their affiliated organizations, or those of the publisher, the editors and the reviewers. Any product that may be evaluated in this article, or claim that may be made by its manufacturer, is not guaranteed or endorsed by the publisher.
